# Multistage Spectral Relaxation Method for Solving the Hyperchaotic Complex Systems

**DOI:** 10.1155/2014/943293

**Published:** 2014-10-16

**Authors:** Hassan Saberi Nik, Paulo Rebelo

**Affiliations:** ^1^Department of Mathematics, Islamic Azad University, Mashhad Branch, Mashhad, Iran; ^2^Departamento de Matemática, Universidade da Beira Interior, 6201-001 Covilhã, Portugal

## Abstract

We present a pseudospectral method application for solving the hyperchaotic complex systems. The proposed method, called the multistage spectral relaxation method (MSRM) is based on a technique of extending Gauss-Seidel type relaxation ideas to systems of nonlinear differential equations and using the Chebyshev pseudospectral methods to solve the resulting system on a sequence of multiple intervals. In this new application, the MSRM is used to solve famous hyperchaotic complex systems such as hyperchaotic complex Lorenz system and the complex permanent magnet synchronous motor. We compare this approach to the Runge-Kutta based ode45 solver to show that the MSRM gives accurate results.

## 1. Introduction

Chaos theory studies the behaviour of dynamical systems that are highly sensitive to initial conditions and have complex and highly unpredictable profiles [1, 2]. Chaotic systems can be observed in a wide variety of applications. In 1982, the complex Lorenz equations were proposed by Fowler et al. [3], which extended nonlinear systems into complex space. After that, some research works in this field have been achieved [4–9]. With in-depth study of complex nonlinear systems, a variety of physical phenomena could be described by the chaotic or hyperchaotic complex systems, for instance, the detuned laser systems and the amplitudes of electromagnetic fields.

The nature of complex chaotic systems precludes the possibility of obtaining closed form analytical solutions of the underlying governing equations. Thus, approximate-analytical methods, which are implemented on a sequence of multiple intervals to increase their radius of convergence, are often used to solve IVPs modelling chaotic systems. Examples of multistage methods that have been developed recently to solve IVPs for chaotic and nonchaotic systems include the multistage homotopy analysis method [10], piecewise homotopy perturbation methods [11, 12], multistage variational iteration method [13], and multistage differential transformation method [14]. Other multistage methods which use numerical integration techniques have also been proposed such as the piecewise spectral homotopy analysis method [15–17] which uses a spectral collocation method to perform the integration process. Accurate solutions of highly chaotic and hyperchaotic systems require resolution over many small intervals. Thus, seeking analytical solutions over the numerous intervals may be impractical or computationally expensive if the solution is sought over very long intervals.

In this paper, we propose a piecewise or multistage spectral relaxation method (MSRM) for solving the hyperchaotic complex systems as an accurate and robust alternative to recent multistage methods. The proposed MSRM was developed using the Gauss-Seidel idea of decoupling systems of equations and using Chebyshev pseudospectral methods to solve the resulting decoupled system on a sequence of multiple intervals. The spectral relaxation method (SRM) was recently proposed in [18, 19].

The rest of the paper is organized as follows. In Section 2, we give a brief description of the proposed MSRM algorithm. In Section 3, we present the numerical implementation of the MSRM on two examples of hyperchaotic complex systems. Finally, the conclusion is given in Section 4.

## 2. Multistage Spectral Relaxation Method

In this section, we give a brief description of the numerical method of solution used to solve the nonlinear hyperchaotic complex. We employ the multistage spectral relaxation method (MSRM) proposed in [19]. The MSRM algorithm is based on a Gauss-Seidel type of relaxation that decouples and linearises the system and the use of spectral collocation method to solve the linearised equations in a sequential manner. For compactness, we express the system of *m* nonlinear first order differential equations in the form

(1) x˙r(t)=∑k=1mαr,kxk(t) +fr[x1(t),x2(t),…,xr−1(t),xr+1(t),…,xm(t)],

subject to the initial conditions

(2) $$\begin{array}{c} x_{r}\left(0\right)=x_{r}^{*},\;r=1,2,\ldots ,m, \end{array}$$

where *x*_*r*_ are the unknown variables and *x*_r_^*^ are the corresponding initial conditions, *α*_*r*,*k*_ are known constant input parameters and *f*_*r*_ is the nonlinear component of the *r*th equation and the dot denotes differentiation with respect to time *t*.

The scheme computes the solution of (1) in a sequence of equal subintervals that makes the entire interval. We define the interval of integration as *Ω* = [0, *T*] and divide it into a sequence of nonoverlapping subintervals *Ω*_*i*_ = [*t*_*i*−1_, *t*_*i*_]  (*i* = 1,2, 3,…, *f*), where *t*_0_ = 0 and *t*_*f*_ = *T*. We denote the solution of (1) in the first subinterval [*t*_0_, *t*_1_] as *x*_*r*_^1^(*t*) and the solutions in the subsequent subintervals [*t*_*i*−1_, *t*_*i*_]  (*i* = 2,3,…, *f*) as *x*_*r*_^*i*^(*t*). For obtaining the solution in the first interval [*t*_0_, *t*_1_], (2) is used as the initial condition. By using the continuity condition between neighbouring subintervals the obtained solution in the interval [*t*_0_, *t*_1_] is used to obtain the initial condition for the next subinterval [*t*_1_, *t*_2_]. This is applied over the *f* successive subintervals; that is, the obtained solution for each subinterval [*t*_*i*−1_, *t*_*i*_] is used to obtain the initial condition for the next subinterval [*t*_*i*_, *t*_*i*+1_]  (*i* = 1,2,…, *f* − 1). Thus, in each interval [*t*_*i*−1_, *t*_*i*_] we must solve

(3) x˙ri=αr,rxri+(1−δrs)∑k=1mαr,kxki +fr[x1i,…,xr−1i,xr+1i,…,xni],

subject to

(4) $$\begin{array}{c} x_{r}^{i}\left(t_{i-1}\right)=x_{r}^{i-1}\left(t_{i-1}\right), \end{array}$$

where *δ*_*rs*_ is the Kronecker delta. As mentioned earlier, the main idea behind the MSRM scheme is decoupling the system of nonlinear IVPs using the Gauss-Seidel idea of decoupling systems of algebraic equations. The proposed MSRM iteration scheme for the solution in the interval *Ω*_*i*_ = [*t*_*i*−1_, *t*_*i*_] is given as

(5)    x˙1,s+1i−α1,1x1,s+1i=α1,2x2,si+α1,3x3,si  +⋯+α1,nxn,si+f1[x1,si,…,xn,si],   x˙2,s+1i−α2,2x2,s+1i=α2,1x1,s+1i+α2,3x3,si  +⋯+α2,nxn,si  +f2[x1,s+1i,x2,si,…,xn,si],   ⋮x˙m,s+1i−αm,mxm,s+1i=αm,1x1,s+1i+⋯+αm,m−1xm−1,s+1i +fm[x1,s+1i,…,xm−1,s+1i,xm,si],

subject to the initial conditions

(6) $$\begin{array}{c} x_{r,s+1}^{i}\left(t_{i-1}\right)=x_{r}^{i-1}\left(t_{i-1}\right),\;r=1,2,\ldots ,m, \end{array}$$

where *x*_*r*,*s*_ is the estimate of the solution after *s* iterations. A suitable initial guess to start the iteration scheme (5) is one that satisfies the initial condition (6). A convenient choice of initial guess that was found to work in the numerical experiments considered in this work is

(7) $$\begin{array}{c} x_{r,0}^{i}\left(t\right)=\{\begin{array}{cc} x_{r}^{*} & \text{if}\;\;i=1,\\ x_{r}^{i-1}\left(t_{i-1}\right) & \text{if}\;\;2\leq i\leq f. \end{array} \end{array}$$

The Chebyshev spectral method is used to solve (5) on each interval [*t*_*i*−1_, *t*_*i*_]. First, the region [*t*_*i*−1_, *t*_*i*_] is transformed to the interval [−1,1] on which the spectral method is defined by using the linear transformation,

(8) $$\begin{array}{c} t=\frac{\left(t_{i}-t_{i-1}\right)\tau }{2}+\frac{\left(t_{i}+t_{i-1}\right)}{2}, \end{array}$$

in each interval [*t*_*i*−1_, *t*_*i*_] for *i* = 1,…, *f*. We then discretize the interval [*t*_*i*−1_, *t*_*i*_] using the Chebyshev-Gauss-Lobatto collocation points [20]:

(9) $$\begin{array}{c} \tau_{j}^{i}=\cos\left(\frac{\pi j}{N}\right),\;j=1,2,\ldots ,N, \end{array}$$

which are the extrema of the *N*th order Chebyshev polynomial:

(10) $$\begin{array}{c} T_{N}\left(\tau \right)=\cos\left(N\cos^{-1}\tau \right). \end{array}$$

The Chebyshev spectral collocation method is based on the idea of introducing a differentiation matrix *D* which is used to approximate the derivatives of the unknown variables *x*_*r*,*s*+1_^*i*^(*t*) at the collocation points as the matrix vector product

(11) $$\begin{array}{c} {\frac{dx_{r,s+1}^{i}}{dt}|}_{t=t_{j}}=\overset{N}{\underset{k=0}{\sum }}D_{jk}x_{r,s+1}^{i}=DX_{r,s+1}^{i},\;j=1,2,\ldots ,N, \end{array}$$

where **D** = 2*D*/(*t*_*i*_ − *t*_*i*−1_) and **X**_*r*,*s*+1_^*i*^ = [*x*_*r*,*s*+1_^*i*^(*τ*_0_^*i*^), *x*_*r*,*s*+1_^*i*^(*τ*_1_^*i*^),…, *x*_*r*,*s*+1_^*i*^(*τ*_*N*_^*i*^)] are the vector functions at the collocation points *τ*_*j*_^*i*^.

Applying the Chebyshev spectral collocation method in (5) gives

(12) ArXr,s+1i=Bri,  Xr,s+1i(τNi−1)=Xri−1(τNi−1),r=1,2,…,m,

with

(13)    Ar=D−αr,rI,  B1i=α1,2X2,si  +⋯+α1,nXn,si+f1[X1,si,…,Xm,si],  B2i=α2,1X1,s+1i+α2,3X3,si  +⋯+α2,mXm,si+f2[X1,s+1i,X2,si,…,Xm,si],  ⋮Bmi=αm,1X1,s+1i+αm,2X2,s+1i+⋯+αm,m−1Xm−1,s+1i  +fm[X1,s+1i,…,Xm−1,s+1i,Xm,si],

where **I** is an identity matrix of order *N* + 1. Thus, starting from the initial approximation (7), the recurrence formula

(14) $$\begin{array}{c} X_{r,s+1}^{i}=A_{r}^{-1}B_{r}^{i},\;r=1,2,\ldots ,m \end{array}$$

can be used to obtain the solution *x*_*r*_^*i*^(*t*) in the interval [*t*_*i*−1_, *t*_*i*_]. The solution approximating *x*_*r*_(*t*) in the entire interval [*t*_0_, *t*_*F*_] is given by

(15) $$\begin{array}{c} x_{r}\left(t\right)=\{\begin{array}{cc} x_{r}^{1}\left(t\right), & t\in \left[t_{0},t_{1}\right]\\ x_{r}^{2}\left(t\right), & t\in \left[t_{1},t_{2}\right]\\ \vdots & \\ x_{r}^{F}\left(t\right), & t\in \left[t_{f-1},t_{f}\right]. \end{array} \end{array}$$

## 3. Numerical Examples

In this section, we consider two examples which demonstrate the efficiency and accuracy of the proposed method. In particular, we use the MSRM algorithm as an appropriate tool for solving nonlinear IVPs; we apply the method to two complex nonlinear chaotic systems.

**Example 1 .**

The hyperchaotic complex Lorenz system can be described as

(16) z˙1=a1(z2−z1)+jz4,z˙2=a2z1−z2−z1z3+jz4,z˙3=12(z1z¯2+z¯1z2)−a3z3,z˙4=12(z1z¯2+z¯1z2)−a4z4,

where *z*_1_ = *x*_1_ + *jx*_2_, *z*_2_ = *x*_3_ + *jx*_4_, *z*_3_ = *x*_5_, *z*_4_ = *x*_6_, $j=\sqrt{-1}$, ${\bar{z}}_{1}$ and ${\bar{z}}_{2}$ are the conjugates of *z*_1_ and *z*_2_. When the parameters are chosen as *a*_1_ = 15, *a*_2_ = 36, *a*_3_ = 4.5, and *a*_4_ = 12, the system (16) is hyperchaotic [21].

Replacing the complex variables in system (16) with real and imaginary number variables, one can get an equivalent system as follows:

(17) x˙1=a1(x3−x1),x˙2=a1(x4−x2)+x6,x˙3=a2x1−x3−x1x5,x˙4=a2x2−x4−x2x5+x6,x˙5=x1x3+x2x4−a3x5,x˙6=x1x3+x2x4−a4x6.

For (17), the parameters *α*_*r*,*k*_ and *f*_*r*_ are defined as

(18) $$\begin{array}{c} \alpha_{1,1}=-a_{1},\;\;\alpha_{1,3}=a_{1},\;\;\alpha_{2,2}=-a_{1},\\ \alpha_{2,4}=a_{1},\;\;\alpha_{2,6}=1,\\ \alpha_{3,1}=a_{2},\;\;\alpha_{3,3}=-1,\;\;\alpha_{4,2}=a_{2},\\ \alpha_{4,4}=-1,\;\;\alpha_{4,6}=1,\\ \alpha_{5,5}=-a_{3},\;\;\alpha_{6,6}=-a_{4},\;\;f_{3}=-x_{1}x_{5},\\ f_{4}=-x_{2}x_{5},\;\;f_{5}=f_{6}=x_{1}x_{3}+x_{2}x_{4}, \end{array}$$

with all other *α*_*r*,*k*_ and *f*_*r*_ = 0 for *r*, *k* = 1,2,…, 6.

**Figure 1:**
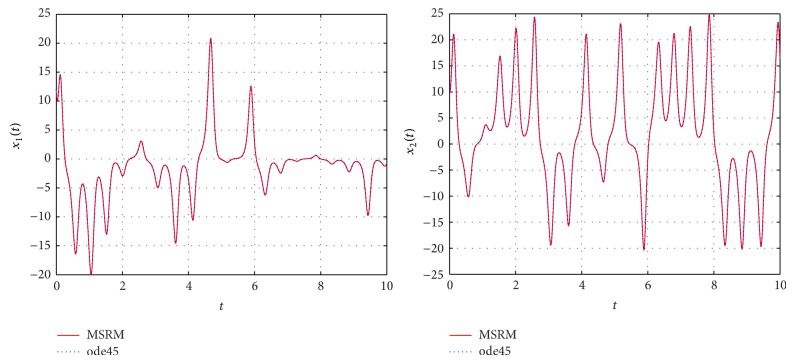
Comparison between the MSRM and ode45 results for the hyperchaotic complex Lorenz system.

**Figure 2:**
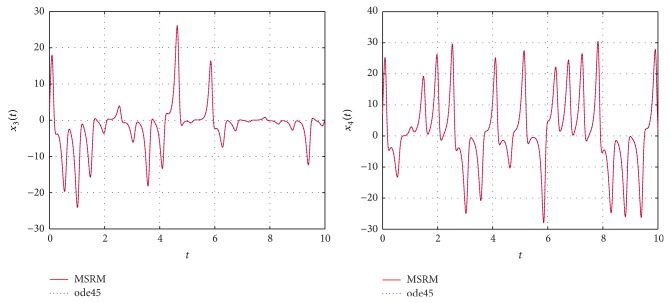
Comparison between the MSRM and ode45 results for the hyperchaotic complex Lorenz system.

**Figure 3:**
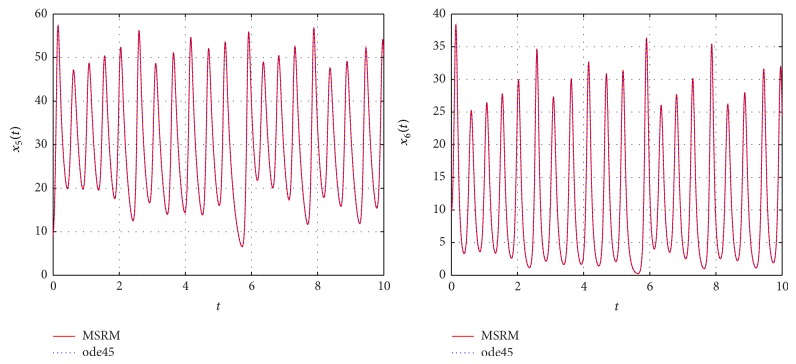
Comparison between the MSRM and ode45 results for the hyperchaotic complex Lorenz system.

**Figure 4:**
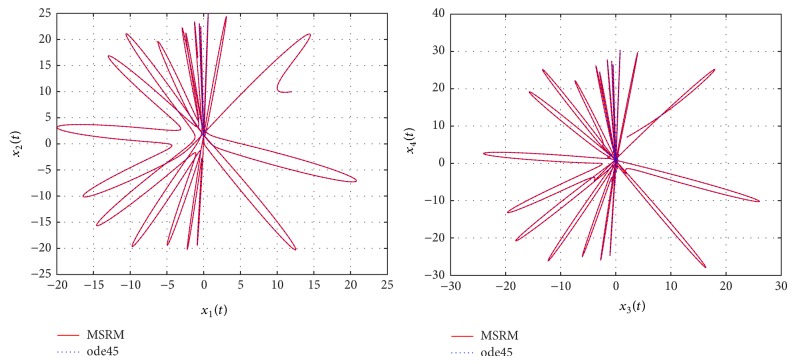
Phase portraits of the hyperchaotic complex Lorenz system.

**Figure 5:**
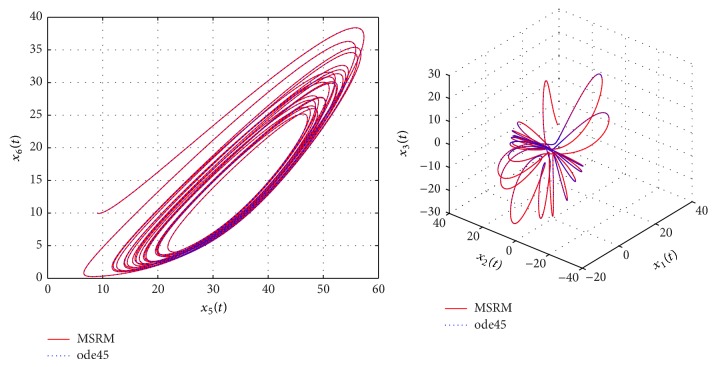
Phase portraits of the hyperchaotic complex Lorenz system.

Through numerical experimentation, it was determined that *N* = 6 collocation points and 5 iterations of the MSRM scheme at each interval were sufficient to give accurate results in each [*t*_*i*−1_, *t*_*i*_] interval. Tables 1 and 2 show a comparison of the solutions of the hyperchaotic complex Lorenz system computed by the MSRM and ode45. In Figures 1, 2, and 3, the MSRM graphical results are also compared with ode45 and good agreement is observed. The MRSM phase portraits in Figures 4 and 5 were also found to be exactly the same as those computed using ode45. This shows that the proposed MSRM is a valid tool for solving the hyperchaotic complex Lorenz system.

**Example 2 .**

State equations of a permanent magnet synchronous motor system in a field-oriented rotor can be described as follows [22, 23]:

(19) diddt=−R1id+ωLqiq+udLd,diqdt=R1iq+ωLdiq+uq−ωΨrLq,dωdt=nqΨrid+np(Ld−Lq)idiq−TL−βωJ,

where *i*_*d*_, *i*_*q*_, and *ω* are the state variables which represent currents and motor angular frequency, respectively; *u*_*d*_ and *u*_*q*_ are the direct-axis stator and quadrature-axis stator voltage components, respectively; *J* is the polar moment of inertia; *T*_*L*_ is the external load torque; *β* is the viscous damping coefficient; *R*_1_ is the stator winding resistance; *L*_*d*_ and *L*_*q*_ are the direct-axis stator inductors and quadrature-axis stator inductors, respectively; Ψ_*r*_ is the permanent magnet flux; and *n*_*p*_ is the number of pole-pairs; the parameters *L*_*d*_, *L*_*q*_, *J*, *T*_*L*_, *R*_1_, Ψ_*r*_, *β* are all positive.

When the air gap is even, and the motor has no load or power outage, the dimensionless equations of a permanent magnet synchronous motor system can be depicted as

(20) z˙1=a(z2−z1),z˙2=bz1−z2−z1z3,z˙3=z1z2−z3,

where *a*, *b* are both positive parameters. If the current in the system (19) is plural and the variables *z*_1_, *z*_2_ in the system (20) are complex numbers, by changing cross coupled terms *z*_1_ and *z*_2_ to conjugate form, Wang and Zhang got a complex permanent magnet synchronous motor system as follows [24]:

(21) z˙1=a(z2−z1),z˙2=bz1−z2−z1z3,z˙3=12(z1z¯2+z¯1z2)−z3,

where *z*_1_ = *x*_1_ + *jx*_2_, *z*_2_ = *x*_3_ + *jx*_4_, *z*_3_ = *x*_5_, $j=\sqrt{-1}$, ${\bar{z}}_{1}$ and ${\bar{z}}_{2}$ are the conjugates of *z*_1_ and *z*_2_. Replacing the complex variables in system (21) with real and imaginary number variables, Wang and Zhang got an equivalent system as follows (see [24]):

(22) x˙1=a(x3−x1),x˙2=a(x4−x2),x˙3=bx1−x3−x1x5,x˙4=bx2−x4−x2x5,x˙5=x1x3+x2x4−x5,

where *a*, *b* are positive parameters determining the chaotic behaviors and bifurcations of system (22). When the parameters satisfy 1 ≤ *a* ≤ 11, 10 ≤ *b* ≤ 20, there is one positive Lyapunov exponent, two Lyapunov exponents of zero, and two negative Lyapunov exponents for system (22), which means system (22) is chaotic [24]. The values of parameters and initial values are *a* = 11, *b* = 20, and *x*_1_(0) = 1, *x*_2_(0) = 2, *x*_3_(0) = 3, *x*_4_(0) = 4, *x*_5_(0) = 5.

For (21), the parameters *α*_*r*,*k*_ and *f*_*r*_ are defined as

(23) $$\begin{array}{c} \alpha_{1,1}=-a,\;\;\alpha_{1,3}=a,\;\;\alpha_{2,2}=-a,\\ \alpha_{2,4}=a,\;\;\alpha_{3,1}=b,\;\;\alpha_{3,3}=-1,\\ \alpha_{4,2}=b,\;\;\alpha_{4,4}=-1,\;\;\alpha_{5,5}=-1,\\ f_{3}=-x_{1}x_{5},\;\;f_{4}=-x_{2}x_{5},\;\;f_{5}=x_{1}x_{3}+x_{2}x_{4}, \end{array}$$

with all other *α*_*r*,*k*_ and *f*_*r*_ = 0 for *r*, *k* = 1,2,…, 5.

**Figure 6:**
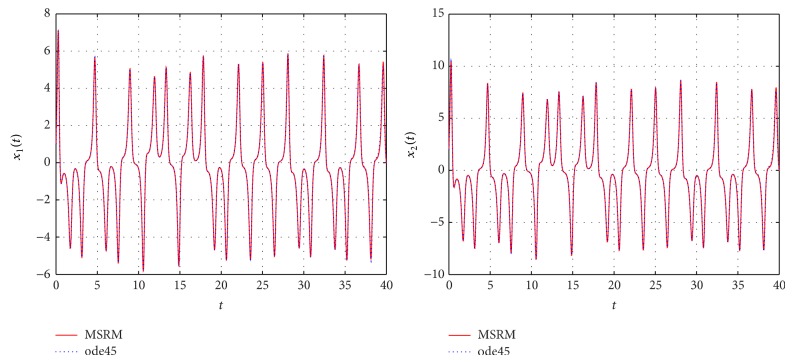
Comparison between the MSRM and ode45 results for the complex permanent magnet synchronous motor.

**Figure 7:**
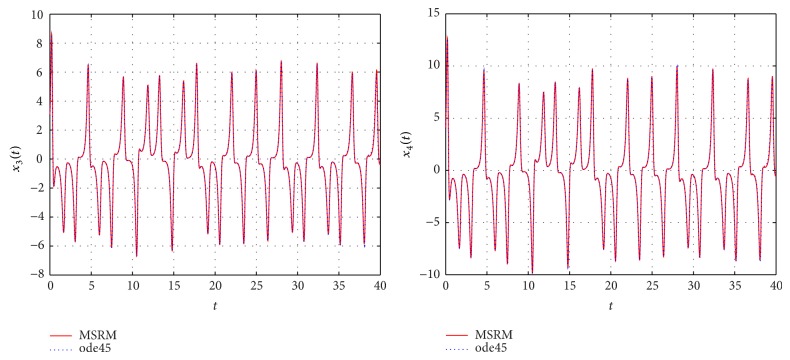
Comparison between the MSRM and ode45 results for the complex permanent magnet synchronous motor.

**Figure 8:**
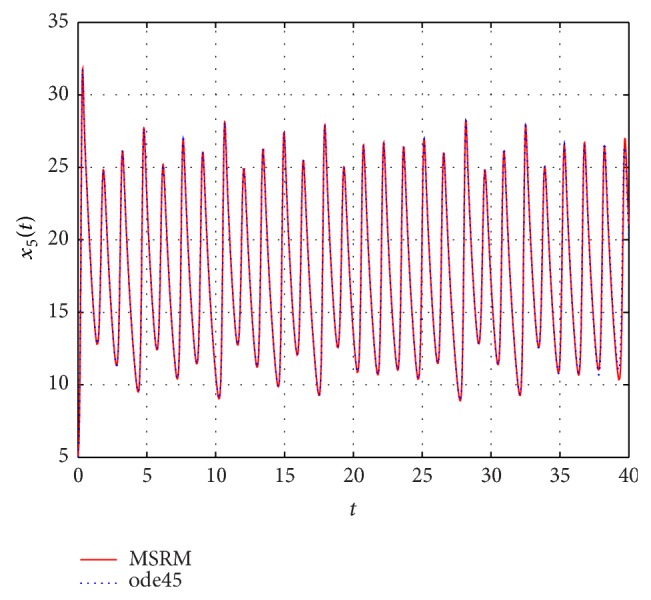
Comparison between the MSRM and ode45 results for the complex permanent magnet synchronous motor.

**Figure 9:**
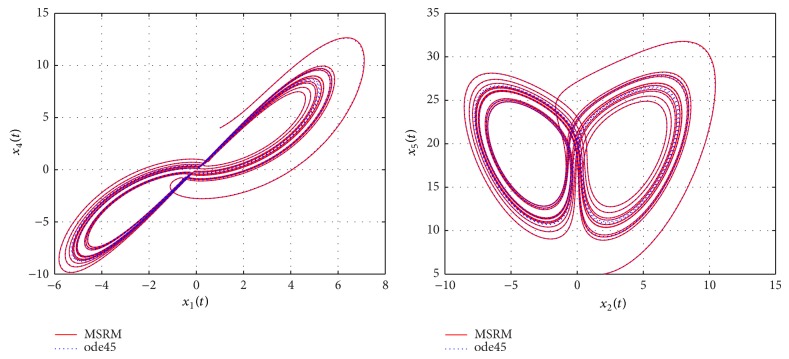
Phase portraits of the complex permanent magnet synchronous motor.

**Figure 10:**
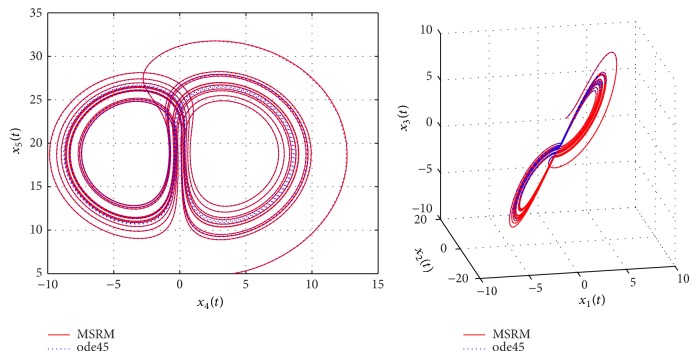
Phase portraits of the complex permanent magnet synchronous motor.

The results obtained were compared to those from the MATLAB inbuilt solver, ode45. The ode45 solver integrates a system of ordinary differential equations using explicit 4th and 5th Runge-Kutta formula. Tables 3 and 4 show a comparison of the solutions of the complex permanent magnet synchronous motor computed by the MSRM and ode45. In Figures 6, 7, and 8, the MSRM graphical results are also compared with ode45 and good agreement is observed. The MRSM phase portraits in Figures 9 and 10 were also found to be exactly the same as those computed using ode45. This shows that the proposed MSRM is a valid tool for solving the complex permanent magnet synchronous motor.

## 4. Conclusion

In this paper, we have applied a spectral method called the multistage spectral relaxation method (MSRM) for the solutions of hyperchaotic complex systems. The proposed MSRM was developed using the Gauss-Seidel idea of decoupling systems of equations and using Chebyshev pseudospectral methods to solve the resulting decoupled system on a sequence of multiple intervals. The proposed MSRM was used to solve the hyperchaotic complex Lorenz system and complex permanent magnet synchronous motor. The accuracy and validity of the proposed method was tested against Matlab Runge-Kutta based inbuilt solvers and against previously published results.

## Figures and Tables

**Table 1:** Numerical comparison between MSRM and o de45 for the hyperchaotic complex Lorenz system.

*t*	*x*_1_(*t*)		*x*_2_(*t*)		*x*_3_(*t*)	
	MSRM	ode45	MSRM	ode45	MSRM	ode45
2	−2.91138	−2.91138	21.73155	21.73155	−3.24491	−3.24491
4	−3.63001	−3.63001	6.52144	6.52144	−6.30884	−6.30884
6	2.80571	2.80571	−2.77638	−2.77638	−2.37099	−2.37099
8	0.01134	0.01134	2.09585	2.09585	−0.14880	−0.14880
10	−0.80219	−0.80219	16.48559	16.48560	−0.06690	−0.06690

**Table 2:** Numerical comparison between MSRM and o de45 for the hyperchaotic complex Lorenz system.

*t*	*x*_4_(*t*)		*x*_5_(*t*)		*x*_6_(*t*)	
	MSRM	ode45	MSRM	ode45	MSRM	ode45
2	23.96851	23.96851	44.32071	44.32071	26.54682	26.54682
4	11.30830	11.30830	14.68007	14.68007	3.25221	3.25221
6	4.65208	4.65208	39.34559	39.34559	12.99055	12.99055
8	−4.99685	−4.99685	33.79560	33.79560	8.02232	8.02232
10	1.98179	1.98179	50.59739	50.59740	24.48234	24.48234

**Table 3:** Numerical comparison between MSRM and o de45 for the complex permanent magnet synchronous motor.

*t*	*x*_1_(*t*)		*x*_2_(*t*)		*x*_3_(*t*)	
	MSRM	ode45	MSRM	ode45	MSRM	ode45
3	−3.85711	−3.85711	−5.66683	−5.66683	−5.20445	−5.20445
10	−0.33729	−0.33729	−0.49554	−0.49554	−0.49104	−0.49104
17	0.12630	0.12631	0.18555	0.18557	0.15550	0.15551
24	0.05091	0.05105	0.07480	0.07501	0.19500	0.19518
31	−2.55034	−2.54878	−3.74694	−3.74465	−0.79819	−0.79326
38	−3.93154	−3.73551	−5.77619	−5.48818	−5.33693	−5.20595

**Table 4:** Numerical comparison between MSRM and o de45 for the complex permanent magnet synchronous motor.

*t*	*x*_4_(*t*)		*x*_5_(*t*)	
	MSRM	ode45	MSRM	ode45
3	−7.64635	−7.64635	15.05932	15.05932
10	−0.72144	−0.72143	10.73663	10.73663
17	0.22846	0.22848	14.25582	14.25583
24	0.28649	0.28675	19.33844	19.33921
31	−1.17270	−1.16545	25.34856	25.35739
38	−7.84098	−7.64855	14.98250	14.03140
